# DNA-launched RNA replicon vaccines induce potent anti-Ebolavirus immune responses that can be further improved by a recombinant MVA boost

**DOI:** 10.1038/s41598-018-31003-6

**Published:** 2018-08-20

**Authors:** Pontus Öhlund, Juan García-Arriaza, Eva Zusinaite, Inga Szurgot, Andres Männik, Annette Kraus, Mart Ustav, Andres Merits, Mariano Esteban, Peter Liljeström, Karl Ljungberg

**Affiliations:** 1 Department of Microbiology, Tumor and Cell Biology, Karolinska Institutet, Stockholm, Sweden; 2 Department of Molecular and Cellular Biology, Centro Nacional de Biotecnología, Consejo Superior de Investigaciones Científicas (CNB-CSIC), Madrid, Spain; 3 Institute of Technology, University of Tartu, Tartu, Estonia; 4 Icosagen Cell Factory OÜ, Ülenurme vald, Tartumaa, Estonia; 5 Department of Microbiology, Public Health Agency of Sweden, Solna, Sweden; 6 Present Address: Department of Biomedical Science and Veterinary Public Health, Virology Unit, Swedish University of Agricultural Sciences, Uppsala, Sweden

## Abstract

There are currently no licensed therapeutic treatment or preventive vaccines against Ebolavirus disease, and the 2013–2016 West African outbreak of Ebolavirus disease spread rapidly and resulted in almost 30,000 cases and more than 11,000 deaths. However, the devastating outbreak has spurred the development of novel Ebolavirus vaccines. Here, we demonstrate that alphavirus-based DNA-launched self-replicating RNA replicon vaccines (DREP) encoding either the glycoprotein (GP) gene or co-expressing the GP and VP40 genes of Sudan or Zaire Ebolavirus are immunogenic in mice inducing both binding and neutralizing antibodies as well as CD8 T cell responses. In addition, antibodies were cross-reactive against another Ebolavirus, although the specificity was higher for the vaccination antigen. DREP vaccines were more immunogenic than recombinant MVA vaccines expressing the same Ebolavirus antigens. However, a DREP prime followed by an MVA boost immunization regimen improved vaccine immunogenicity as compared to DREP and MVA homologous prime-boost immunizations. Moreover, we show that a bivalent approach targeting both Sudan and Zaire Ebolavirus can be employed without significant loss of immunity. This opens for further investigation of a pan-Ebolavirus or even a pan-filovirus vaccine.

## Introduction

Ebolavirus, the causative agent of Ebolavirus Disease (EVD), was discovered in 1976 during outbreaks in Zaire and Sudan^1^. The Ebolavirus genus consists of 5 distinct species; Zaire (EBOV), Sudan (SUDV), Bundibugyo (BDBV), Taï Forest (TAFV) and Reston (RESTV) Ebolaviruses. Ebolavirus causes sporadic outbreaks of hemorrhagic fever, primarily in east and central Africa, and is suspected to be transmitted to humans from fruit bats, which are believed to be the putative primary reservoir, or through intermediate hosts like non-human primates (NHP). Human-to-human transmission occurs via exchange of body fluids and secretions such as blood, semen, saliva etc. Pathogenicity in humans varies from the more aggressive and highly pathogenic EBOV to RESTV, which, although potentially lethal in NHP, appears to be non-pathogenic in humans^2^. The most severe Ebolavirus epidemic thus far started in December 2013, and rapidly spread from Guinea to Liberia and Sierra Leone^3^. This outbreak, caused by the EBOV Makona variant, lasted until March 2016 and counted a total of 28,616 confirmed, probable and suspected cases and 11,310 deaths (WHO Situation Report Ebola Virus Disease, 10 June 2016; http://who.int/csr/disease/ebola/en/).

Ebolavirus is an enveloped virus with a characteristic filamentous shape, belonging to the *Filoviridae* family. The genome consists of a negative-sense single-stranded RNA molecule of 19 kb containing seven genes: nucleoprotein (NP), virion protein (VP) 35, VP40, glycoprotein (GP), VP30, VP24, and RNA-dependent RNA polymerase (L)^1^. The Ebolavirus surface GP is responsible for virus attachment and penetration into the host cell, and is the main target for antibodies to bind and neutralize the virus. Polymerization of the VP40 links the viral nucleocapsid to the viral envelope and recruits GP, thus driving budding and formation of filamentous virus-like particles (VLP)^4^.

Several vaccine candidates have advanced into safety and immunogenicity phase I/II clinical trials and one candidate also into a phase III efficacy clinical trial. The most advanced candidates are based on viral vectors such as replicating Vesicular Stomatitis Virus (rVSV-ZEBOV)^5^, replication defective chimpanzee adenovirus type 3-vector (ChAd3-EBOZ)^6^, replication defective adenovirus type 26-vector vaccine (Ad26-ZEBOV) and Modified Vaccinia Virus Ankara (MVA-BN Filo)^7–9^ encoding the EBOV GP. In the rVSV-ZEBOV vaccine, the VSV-GP has been replaced by the GP from the EBOV Kikwit-95 variant. This replacement of GP attenuates the pathogenicity of the VSV while allowing the vaccine to enter permissive cells via the interaction between the EBOV GP and host cell receptors^10^. MVA-BN Filo is a multivalent vaccine encoding GP from EBOV, SUDV, TAFV and Marburg virus (MARV).

The rVSV-ZEBOV has demonstrated efficacy in a study during the West African outbreak comparing 7651 exposed individuals, where 4123 were assigned to immediate vaccination and 3528 people were assigned to delayed vaccination^11^. There were no Ebolavirus cases in the group receiving the vaccine immediately compared to 16 Ebolavirus cases in the delayed group thus demonstrating protective efficacy. The ChAd3-EBOZ as well as the Ad26-ZEBOV in prime-boost combinations with MVA-BN Filo have demonstrated safety and immunogenicity in phase I and II clinical trials^7,12^, with MVA-BN Filo showing particular potency as a boosting agent to recombinant adenovirus-vectored Ebolavirus vaccines^7–9^. However, despite the considerable advances towards a licensed Ebolavirus vaccine, it is yet unknown whether any of the present candidates will meet all the desirable requirements for a vaccine against Ebola such as being safe with broad and durable immunity. Although both the VSV and adenovirus vectors have passed safety testing in clinical trials, there are still some concerns that rare events are unnoticed in clinical trials with limited numbers of volunteers. For instance, rVSV-ZEBOV is a replication competent vector, and there is a risk of reversion to a pathogenic phenotype, especially in immune compromised individuals. Moreover, there are concerns that rVSV vectors used for vaccinations in humans might spill over to and cause disease in livestock. Another concern is that the neurotoxicity of VSV might be transferred to replication competent VSV vectors. Indeed, there are reports of rVSV neurotoxicity in non-human primates^13^. For adenoviral vectors, determining the safe dose is necessary since high doses of adenovirus particles are toxic to NHPs and humans^14^.

We have generated alphavirus-based DNA-launched replicons (DREP) encoding Ebolavirus antigens that can be delivered as naked DNA vaccines. Upon delivery into host cells, the DREP vaccine will launch an RNA replicon, which is a self-amplifying recombinant RNA molecule. The encoded replicase will amplify the replicon in the cytoplasm as well as transcribe an mRNA encoding the Ebolavirus antigen from an internal viral promoter in the replicon. Such DREP Ebolavirus vaccines offer several advantages; they can be easily and rapidly constructed using synthetic cDNA in response to an emerging threat, and production times are short. Furthermore, DNA-launched replicon vaccines have demonstrated superior immunogenicity over traditional DNA vaccines^15–17^. Replicon vectors have inherent adjuvant properties that are linked to their self-amplifying RNA and replicase activity which generates RNA intermediates that stimulate pattern recognition receptors (PRR) including the endosomal TLR3, TLR7, and TLR8^18,19^, and the cytoplasmic MDA-5^20^, RIG-I^20^ and PKR^21,22^. PRR stimulation results in induction of a type I interferon (IFN) response, host cell translational shutoff and induction of apoptosis thereby promoting cross-priming of antigen epitopes on MHC class I molecules^23–26^. Apoptosis-induction of the transfected antigen-producing cell eliminates the risk of chromosomal integration and the unlikely event of subsequent malignant transformation. Thus, DREP vaccines combine the safety features of conventional DNA vaccines that have proved safe in multiple clinical trials, with adjuvant properties of viral vectors. DREP vaccines need considerably lower doses than conventional DNA vaccines to be immunogenic^15,16,27,28^, especially when combined with electroporation as the mode of delivery. One DREP vaccine that was tested in NHP, demonstrated safety and immunogenicity at doses significantly lower than what is normally used for conventional DNA vaccines^27^. Thus, production of DREP vaccines might be considerably cheaper as compared to conventional DNA vaccines. In this study, we describe the immunogenicity of DREP vaccines encoding the GP and GP-VP40 genes from SUDV and EBOV Ebolavirus species in different prime-boost immunization regimens.

## Results

### Generation of DREP-Ebolavirus vaccine constructs expressing GP or GP-VP40 from SUDV and EBOV

**Figure 1:**
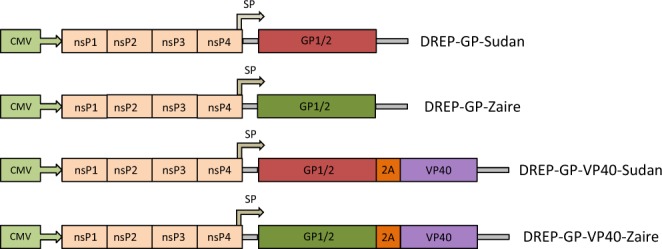
DREP Ebolavirus constructs generated in this study. The different DREP Ebolavirus constructs that have been generated for this study are DREP-GP-Sudan (D-GP-S), DREP-GP-Zaire (D-GP-Z), DREP-GP-VP40-Sudan (D-GP-VP40-S) and DREP-GP-VP40-Zaire (D-GP-VP40-Z), expressing either the GP or GP and VP40 genes from SUDV or EBOV. A description of the 2 A peptide is included in the Methods section. In the DREP plasmids the GP or GP-2A-VP40 genes are placed under the alphaviral subgenomic promoter (SP). The full-length replicon is placed under the cytomegalovirus promoter (CMV).

**Figure 2:**
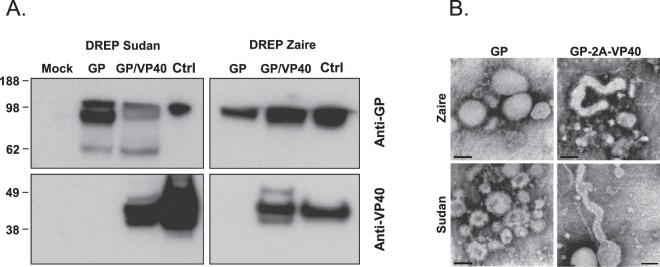
Analysis of Ebolavirus GP and VP40 expression. (**A**) BHK-21 cells were transfected with DREP expressing either the GP or GP and VP40 genes from SUDV or EBOV. Cell lysates were collected and run on an SDS-PAGE. Western blotting was performed using anti-GP and anti-VP40 antibodies. EBOV and SUDV VLPs (Ctrl) were used as positive controls. The blots have been cropped and full blots are available in Supplementary Information. (**B**) Replicon-driven expression of SUDV and EBOV GP and GP-VP40 VLPs was analyzed by electron microscopy (EM) from cell culture supernatants from cells expressing GP and GP-VP40. EM micrographs of VLP preparations reveal pleomorphic VLPs made from GP expressing cells (left panels), and filamentous VLP structures made from GP-VP40 expressing cells (right panels). Black bars indicate 100 nm.

We generated DNA-launched replicons encoding the Ebolavirus GP or GP-VP40 genes from SUDV and EBOV (referred to as D-GP-S, D-GP-Z, D-GP-VP40-S and D-GP-VP40-Z) (Fig. 1). It has been shown that cells expressing Ebolavirus GP or GP and VP40 proteins produce Ebolavirus VLP, with GP-expressing cells giving rise to pleomorphic budding particles whereas cells co-expressing GP and VP40 produce filamentous filovirus like particles^29^. We hypothesized that DREP Ebolavirus constructs expressing either GP or GP plus VP40 may differ in their immunogenic properties. Transfection with 2.5 μg of the D-GP-S, D-GP-Z, D-GP-VP40-S and D-GP-VP40-Z constructs into 90% confluent BHK-21 cells lead to Ebolavirus antigen expression, as determined by Western blot (Fig. 2a). GP and VP40 proteins of expected sizes were detected in cell lysates of D-GP and D-GP-VP40 transfected cells analyzed using Ebolavirus antigen- and species-specific antibodies (Fig. 2a). However, we could not detect any Ebolavirus-like particles from DREP transfected cell supernatants by Western blotting, possibly due to insufficient transfection frequency (data not shown). Thus, we next analyzed supernatants from cell cultures transduced at a high multiplicity of infection with GP- or GP-2A-VP40-expressing viral replicons by electron microscopy. Cells expressing GP only produced pleomorphic particles whereas cells expressing GP and VP40 produced both filovirus-like and pleomorphic structures with GP-resembling spikes on the surface (Fig. 2b).

### The DREP-GP and DREP-GP-VP40 vaccine candidates are immunogenic in mice

**Figure 3:**
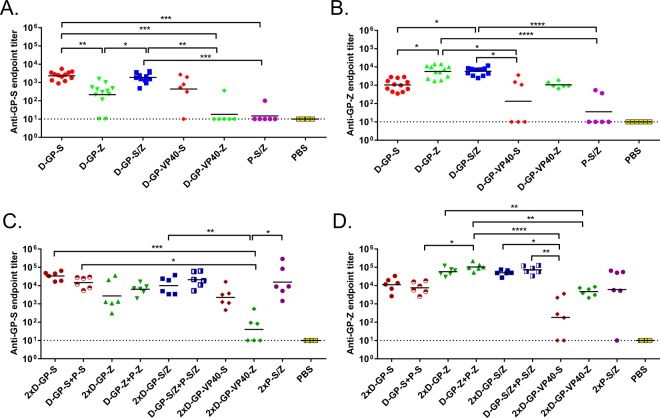
Anti-GP Ebolavirus antibody responses. Anti-SUDV-GP (**A**,**C**) and anti-EBOV-GP (**B**,**D**) antibody endpoint titers were determined in immunized mice after prime (**A**,**B**) and after boost (**C**,**D**). Mice were immunized with 5 µg of DREP Ebolavirus constructs (D-GP-S, D-GP-Z, D-GP-S/Z; n = 12/group, D-GP-VP40-S, D-GP-VP40-Z; n = 6/group) or GP protein (P-S/Z; n = 6/group) and bled 21 days after the immunization and individual serum samples were assayed by ELISA using plates coated with SUDV or EBOV GP (A and B, respectively). In panels C and D, mice (n = 6/group) were primed as above and boosted on day 28 and blood was collected on day 49 individual serum samples were assayed by ELISA using plates coated with SUDV or EBOV GP (C,D, respectively). Statistical analysis was performed using Kruskal Wallis test followed by Dunn’s test for multiple comparisons, *P < 0.05; **P < 0.01; ***P < 0.001; ****P < 0.001. Comparisons that were non significant are not indicated.

To evaluate the immunogenicity of the D-GP and D-GP-VP40 Ebolavirus vaccine candidates, mice were immunized with 5 μg of D-GP from SUDV or EBOV (D-GP-S and D-GP-Z, respectively) or 5 μg of D-GP-VP40-S or D-GP-VP40-Z in homologous prime-boost regimens. For comparison, we also performed immunizations with 5 μg of recombinant GP protein of SUDV and EBOV (designated P-S and P-Z, respectively) formulated with the adjuvant MPLA. Additionally, we performed immunizations with D-GP and protein immunogens in heterologous prime-boost regimens. Mice that received the identical priming immunization are represented as one group before booster immunizations with different vaccine candidates were administered. Experimentally, the animals were treated as independent groups and randomized prior to the start of the experiment. The levels of binding anti-Ebolavirus antibodies against SUDV and EBOV were determined by ELISA after prime (Fig. 3a,b) and after boost (Fig. 3c,d).

One immunization with D-GP-S induced robust anti-SUDV-GP endpoint antibody titers having a geometric mean of 2300 while the D-GP-Z immunization resulted in anti-EBOV-GP antibody titers of around 5700. Booster immunization with D-GP-S or D-GP-Z augmented the endpoint antibody titers about 10-fold (to 33500 and 57000, respectively). A prime with the D-GP vaccines could also be boosted with the GP protein vaccine to similar levels elicited by homologous D-GP vaccination. In contrast, priming animals with a combination of P-S and P-Z, did not result in any significant antibody titers. A homologous P-S/P-Z booster immunization, however, increased the antibody titers substantially. Surprisingly, mice immunized with D-GP-VP40 (D-GP-VP40-S or D-GP-VP40-Z) elicited significantly lower antibody responses compared to D-GP (D-GP-S or D-GP-Z) after a single immunization. A homologous booster immunization increased the responses, but only to levels comparable to levels obtained after one immunization with D-GP.

### The Ebolavirus GP-specific antibody responses induced by the DREP Ebolavirus vaccine candidates are cross-reactive

The sera obtained from the immunizations were also tested for cross-reactivity to the non-homologous Ebolavirus species by ELISA, i.e. sera from mice immunized with constructs encoding SUDV GP were tested for binding of EBOV GP and vice versa. In all cases we observed clear reactivity against the GP from the heterologous Ebolavirus species albeit the responses remained lower than against the GP from the homologous Ebolavirus species. This is illustrated by the presence of antibodies binding to SUDV GP in sera collected from D-GP-Z immunized mice (Fig. 3a,c) and by antibodies binding to EBOV GP in sera collected from D-GP-S immunized mice (Fig. 3b,d). Typically, D-GP cross-reactive titers were about one order of magnitude lower as compared the titers induced by a homologous vaccination regimen, both after one and two immunizations.

### The DREP Ebolavirus vaccine candidates can be used as a bivalent Ebolavirus vaccine

The EBOV and SUDV species are currently the two most relevant Ebolavirus targets for vaccination, although protection against RESTV, TAFV and BDBV might also be important. Ideally, an Ebolavirus vaccine should protect against all or the most common or lethal Ebolavirus species. Thus, we tested whether we could elicit immune responses to both SUDV and EBOV GP antigens using a bivalent vaccination approach immunizing mice with a combination of 2.5 µg each of D-GP-S and D-GP-Z (referred to as D-GP-S/Z). Since the D-GP-VP40 constructs were found to be considerably less immunogenic, they were not tested in the bivalent immunization regimen.

One immunization with bivalent D-GP-S/Z induced high antibody responses to both SUDV and EBOV GP antigens (Fig. 3a,b, respectively). The levels were not different to the levels induced by the single Ebolavirus species vaccine regimens to the homologous antigens after prime. After a second immunization with bivalent D-GP-S/Z, antibody titers were boosted to levels comparable to the responses induced by the single vaccine regimens to their respective antigen (Fig. 3c,d). Thus, specific antibody responses to both SUDV and EBOV GP antigens were induced by the bivalent D-GP-S/Z vaccine that were similar to and not statistically different from the responses induced by D-GP-S or D-GP-Z.

Furthermore, the D-GP-S/Z prime was also boosted using a bivalent Ebolavirus GP protein immunogen (P-GP-S/Z), that resulted in antibody titers that were not different to those obtained from the corresponding homologous D-GP-S followed by P-S and D-GP-Z followed by P-Z immunizations (Fig. 3c,d, respectively). These results demonstrate that the bivalent vaccine approach can induce strong immune responses against both Ebolavirus species without any apparent immunodominance or decrease in titer.

### The DREP-GP vaccine candidates favor Th1 type antibody profiles

**Figure 4:**
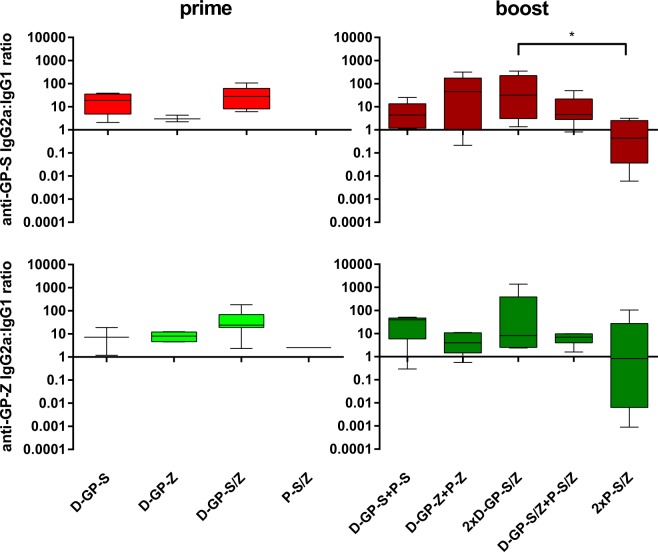
Anti-GP Ebolavirus antibody isotype analysis. Sera from mice primed with D-GP constructs (D-GP-S, D-GP-Z, D-GP-S/Z) or with P-S/Z, and from mice boosted with GP or DREP as indicated (n = 6/group) were analyzed by ELISA for anti-GP EBOV and SUDV antibody isotypes IgG2a and IgG1. Results are presented as IgG2a:IgG1 endpoint antibody titer ratios against GP-S (top panels) or GP-Z (bottom panels). A ratio above 1 indicates a Th1 biased response. Statistical analysis was performed using Kruskal Wallis test followed by Dunn’s test for multiple comparisons, *P < 0.05.

The production of IgG2a and IgG1 are associated with production of cytokines consistent with a Th1 or a Th2 profile, respectively and can be used as an indication of a predominantly Th1 or Th2 biased response^30^. We have previously reported that DREP vaccines predominantly drive Th1 type responses^31,32^, but also noted that T helper profiles are partially antigen dependent (manuscript in preparation). Furthermore, it has been shown that different antibody isotypes may have different effects on Ebolavirus infection in experimental models^33^, and that antibodies corresponding to the IgG2a isotype in mice (i.e. having antibody-dependent cellular cytotoxicity [ADCC] and complement dependent cytotoxicity [CDC] activity) might be beneficial for raising protective responses^34^. Thus, to evaluate how the different DREP-GP immunogens affected Th1 and Th2 induction, we determined the ratio of IgG2a and IgG1 antibody titers against GP EBOV and SUDV antigens (Fig. 4). We observed that mice immunized with one or two doses of the D-GP constructs induced higher IgG2a antibody titers compared to IgG1, corresponding to a Th1 biased response. In contrast, immunization with two doses of the GP protein preparations was characterized by higher IgG1 responses than IgG2a indicating a Th2 type response. In Fig. 4, the ratios between antibody titers in different groups of vaccinated mice (n = 6) are displayed. In a direct comparison, the anti-GP EBOV and SUDV IgG2a/IgG1 ratios were significantly higher in animals immunized with the 2xD-GP-S/Z regimen as compared to the 2x-P-S/Z regimen (Kruskal-Wallis test). Test for multiple comparisons further corroborated this finding for anti-GP-SUDV antibodies. Furthermore, the anti-GP EBOV and SUDV IgG2a/IgG1 ratios from sera from the 2xD-GP-S/Z immunized mice were significantly different from a balanced IgG2a/IgG1 response (ratio of 1; Wilcoxon Signed Rank Test). Interestingly, when a D-GP prime was followed by GP protein boost, the higher IgG2a/IgG ratio induced by the D-GP prime was maintained.

### The DREP Ebolavirus vaccine candidates induce neutralizing antibodies against Ebolavirus

**Figure 5:**
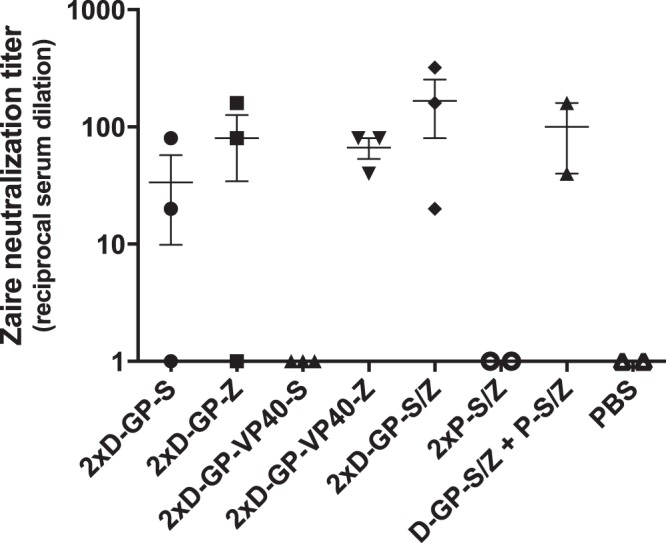
Neutralizing antibody analysis. Selected sera from immunized mice (2–3 serum samples displaying the highest anti-GP EBOV binding antibody titers from each group) were used in a CPE-based EBOV neutralization assay. Sera in two-fold serial dilutions were mixed with 50 pfu of EBOV for 1 h and then applied to Vero cells. Neutralization indicates no detectable CPE in cell cultures in any of the replicate wells over the 10-day assay period, and the neutralization titer is the highest dilution without detectable CPE in the cell culture.

Although binding antibodies may be indicative of protective immunity, neutralizing antibodies are considered crucial for protection against Ebolavirus infection^6,35^. We had the possibility to analyze neutralization of infectious EBOV in a BSL-4 safety laboratory for a limited number of samples (2–3 serum samples displaying the highest ELISA titers were selected for this analysis from each group), and we therefore assessed whether our DREP Ebolavirus and GP protein vaccine candidates were able to induce neutralizing antibodies at 3 weeks post boost. In order to process as many samples as possible, we chose an assay with the readout based on CPE that can be performed in a 96 well format, rather than the more laborious but customary PRNT50 assay. The results indicated that the D-GP-Z and D-GP-VP40-Z vaccine candidates induced neutralizing antibodies against the EBOV species (Fig. 5). Interestingly, sera from the D-GP-S immunized mice, while having lower anti-GP EBOV binding antibody titers (see Fig. 3d), also displayed neutralizing activity against EBOV demonstrating cross-neutralization. However, the D-GP-VP40-S vaccine construct did not elicit cross-neutralizing antibodies. This observation was consistent with induction of only low levels of cross-reactive anti-GP EBOV binding antibodies elicited by this vaccine candidate (see Fig. 3d). Consequently, the D-GP-VP40 constructs were not further analyzed in this work. Interestingly, the neutralization obtained in the group immunized with the bivalent D-GP-S/Z vaccine was comparable to D-GP-Z. In contrast, immunization with the purified GP protein preparation did not induce any neutralizing antibodies although the anti-GP binding antibody titers were similar to those induced by the DREP Ebolavirus vaccines (see Fig. 3c,d). A summary of the relationship between neutralizing and binding antibody titers is given in Table 1.

**Table 1:** Relationship between binding antibody titers and neutralizing antibody titers against EBOV.

Immunization	Average ELISA titer	Average NT titer
2xD-GP-S	16623	34
2xD-GP-Z	79974	80
2xD-GP-VP40-S	2007	<limit of detection
2xD-GP-VP40-Z	6289	67
2xD-GP-S/Z	62906	167
2xP-S/Z	52992	<limit of detection
D-GP-S/Z + P-S/Z	107274	100
PBS	10	<limit of detection

### A mixed modality DREP-GP prime followed by MVA-GP boost regimen induces high levels of Ebolavirus GP-specific antibodies

**Figure 6:**
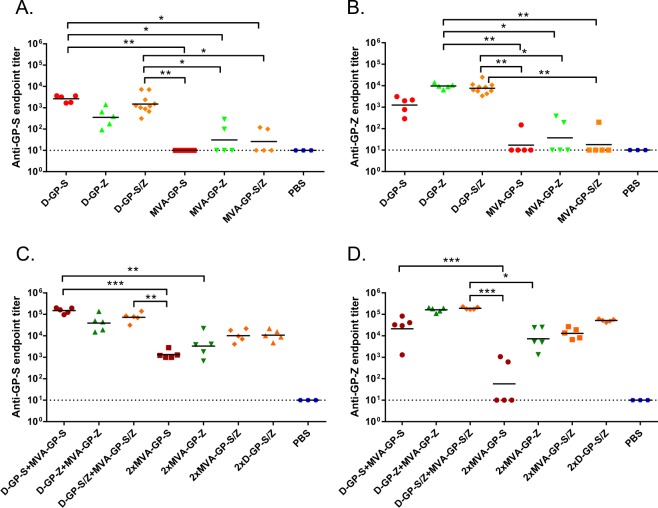
Anti-GP Ebolavirus antibody responses after heterologous DREP-GP/MVA-GP prime-boost immunizations. Serum antibody responses to SUDV (**A**,**C**) and EBOV (**B**,**D**) after prime-boost immunizations in mice. Animals were primed with 5 µg of D-GP-S (n = 5), D-GP-Z (n = 5), D-GP-S/Z; (n = 10), or with 1 × 10^7^ pfu of MVA-GP-S (n = 5), MVA-GP-Z (n = 5) or MVA-GP-S/Z (n = 5) (**A** and **B**) and were boosted with 5 µg of D-GP-S/Z (n = 5) or 1 × 10^7^ pfu of MVA-S (n = 5), MVA-Z (n = 5) or MVAS/Z (n = 5) (**C**,**D**). Animals were immunized on days 1 (prime; 1x) and 28 (boost; 2x) and blood was collected on days 21 (primary response) (**A**,**B**) and 49 (booster response) (**C**,**D**), and sera were assayed by ELISA. Statistical analysis was performed using Kruskal Wallis test followed by Dunn’s test for multiple comparisons, *P < 0.05; **P < 0.01; ***P < 0.001. Comparisons that were non significant are not indicated.

We have previously demonstrated that mixed modality prime-boost regimens consisting of DREP prime followed by MVA boost are highly immunogenic in mice^31,32^ and NHP^27^. Thus, we next tested the hypothesis that D-GP prime immune responses could be further boosted by MVA-GP, an MVA vector expressing GP from SUDV or EBOV species (Fig. 6). Mice that received the identical priming immunization are represented as one group before booster immunizations with different vaccine candidates were administered. Experimentally, the animals were treated as independent groups and randomized prior to the start of the experiment. The SUDV or EBOV GP-specific antibody titers elicited after a D-GP-S, D-GP-Z and D-GP-S/Z prime (Fig. 6a,b, respectively) were boosted about 20–40 fold by the corresponding MVA-GP boost (Fig. 6c,d), compared to a 10-fold increase in the D-GP prime-boost regimen (compare to Fig. 3), demonstrating the potential to further boost the induction of binding anti-GP antibodies by a D-GP priming followed by a MVA-GP boosting immunization protocol. On its own, MVA-GP prime was significantly less immunogenic than D-GP (Fig. 6a,b), although two MVA-GP immunizations resulted in GP-specific antibody titers similar to those obtained after a single DREP immunization. Interestingly, two doses of the bivalent MVA-GP-S/Z elicited higher GP-specific antibody titers than the monovalent vaccine, and after the second immunization the titers were comparable to those of mice immunized two times with D-GP-S/Z.

### Immunization with DREP-GP and MVA-GP induce Ebolavirus-specific T cell responses

**Figure 7:**
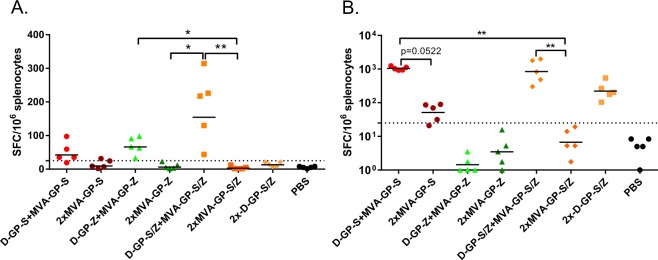
Analysis of GP-specific T cell responses. T cell responses were analyzed by ELISpot after restimulation of splenocytes with the CD8 + restricted peptides LYDRLASTV (**A**) present in both SUDV and EBOV GP or RPHTPQFLF (**B**) from the SUDV GP protein. Mice were primed and boosted with 5 µg of D-GP-S/Z (2.5 μg of each) constructs or 1 × 10^7^ pfu of MVA-GP-S/Z vaccine candidates as indicated. Animals were immunized on days 1 (prime) and 28 (boost) and spleens were collected 21 days after boost. The dotted line represents the cutoff for a positive response (25 SFC per million splenocytes). Statistical analysis was performed comparing all groups except PBS immunized controls using Kruskal Wallis test followed by Dunn’s test for multiple comparisons, *P < 0.05; **P < 0.01. Comparisons that were non-significant are not indicated. Animals immunized with the EBOV virus GP were excluded from the statistical multiple comparison analysis for the results with the SUDV derived peptide in panel B, since they did not encode the epitope used for restimulation.

While antibodies are the best correlate of protection for Ebolavirus infection^36^, CD8 + T cells may blunt the infection and contribute to faster recovery^37^. Thus, we analyzed the induction of Ebolavirus GP-specific T cell responses in splenocytes from mice immunized with the bivalent DREP-GP-S/Z and MVA-GP-S/Z vaccine candidates by IFN-γ ELISpot (Fig. 7). We tested the reactivity to two CD8 + T cell restricted epitopes^38^: the LYDRLASTV peptide present in both SUDV and EBOV GP (Fig. 7a) and the SUDV GP peptide RPHTPQFLF (Fig. 7b) in mice that were primed with D-GP or MVA-GP and boosted with D-GP or MVA-GP.

Responses in splenocytes restimulated with the CD8 + T cell restricted LYDRLASTV peptide were generally low but detectable in mice primed with D-GP and boosted with MVA-GP. The heterologous prime-boost regimen induced significantly higher frequencies of IFN-γ secreting cells than a homologous prime-boost immunization with MVA-GP, which gave very low to undetectable responses. Clearly, the bivalent D-GP-S/Z + MVA-GP-S/Z prime-boost immunization elicited the strongest responses against this epitope. Also when splenocytes were restimulated with the more potent SUDV-derived CD8 + T cell restricted RPHTPQFLF epitope, the heterologous D-GP + MVA-GP prime-boost immunization schedule elicited significantly higher frequencies of IFN-γ secreting cells. As expected, the D-GP-S + MVA-GP-S was the most potent inducer of GP-specific CD8 + T cells. In contrast, homologous D-GP-S/Z prime-boost immunization generated moderate GP-specific CD8 + T cell responses, whereas MVA-GP-S/Z did not elicit detectable frequencies of IFN-γ secreting cells.

## Discussion

Despite promising results of viral vectored vaccine candidates against Ebolavirus such as the rVSV-ZEBOV^11^, ChAd3-EBOZ^6^ and ChAd3-EBOZ or Ad26-ZEBOV prime followed by MVA-BN Filo boost^7–9^, there are still concerns regarding both safety and durability of the immune responses. For instance, the ChAd3-EBOZ vaccine has reported waning immunity dropping to only 50% protection ten months after immunization in NHP^6^. Consequently, it is necessary to develop additional vaccine candidates, as different vaccine approaches and platforms may have their distinct merits, and may work synergistically in heterologous prime-boost regimens. The ideal vaccine for protection against Ebolavirus infection and/or disease is a vaccine that is safe, immunogenic, thermo-stable and inexpensive. Moreover, it should induce long-term protection to multiple Ebolavirus species after a single immunization. The vaccines tested in efficacy trials only target EBOV, although it might be desirable to also induce immunity to other filoviruses of epidemic potential. Certainly, immunization against one Ebolavirus may confer partial protection to other Ebolavirus species, but given the high degree of amino acid diversity between the GP sequences of different Ebolavirus, a species-specific vaccine is likely to be more efficient^39^. The ChAd3-EBOZ vaccine used a bivalent approach initially, but the SUDV was excluded from the vaccine to advance production in response to the 2013–2016 West African outbreak^40^. The multivalent MVA-BN Filo has been tested in clinical trials in different prime-boost regimens with ChAd3-EBOZ or Ad26-ZEBOV in order to broaden the immune responses^7–9^, although induction of antibodies specific for SUDV, TAFV or MARV GP were not formally demonstrated in the clinical studies.

In the present study, we demonstrate that DREP Ebolavirus vaccines elicit robust Ebolavirus-specific humoral and cellular immune responses that can be further increased by booster immunizations with the same vector or with a heterologous vaccine such as purified GP protein or an MVA-based vaccine expressing GP. Although cellular immune responses certainly contribute to recovery after infection and even have been shown to alone mediate protection in some studies^37^, antibody levels specific for Ebolavirus provide a useful correlate of protection in mice, guinea pigs and NHP^36^, with neutralizing antibodies being considered as the main correlate of protection against EVD^6,35^. This is supported by the use of monoclonal neutralizing antibodies such as ZMapp as post exposure prophylaxis to treat an established Ebolavirus infection in NHP^41^. ZMapp was used to treat Ebolavirus infected individuals during the West African outbreak, and 28 of 36 treated patients survived compared to 22 of 35 in the standard of care comparator group. However, the seemingly beneficial effect of ZMapp did not meet the prespecified statistical threshold for efficacy^42^. Larger trials may be needed to determine the treatment efficacy and therapeutic window. Here, we show that immunization with DREP encoding GP elicited high titers of Ebolavirus-specific antibodies. Moreover, GP-encoding vaccines derived from SUDV elicited cross-reactive antibodies that bind EBOV GP and vice versa. The cross-reactive binding titers were, however, lower than the titers to GP of the homologous Ebolavirus species. In humans, Ebolavirus infection has been shown to induce cross-reactive antibodies that target conserved epitopes on the GP surface and are capable of neutralizing multiple Ebolavirus species^43–46^, and in mice some cross-reactive monoclonal antibodies can protect against both SUDV and EBOV infection^47^. In a virus neutralization assay using the wild type EBOV, we demonstrated the ability of D-GP vaccine-elicited antibodies to both neutralize and cross-neutralize EBOV. However, two immunizations with D-GP-VP40-S elicited EBOV cross-binding antibody titers that were 1–2 orders of magnitude lower than two immunizations with D-GP-S, and failed to neutralize EBOV. Thus it appears that a threshold antibody titer has to be reached in order for a serum to display any significant neutralization in our assay. Nevertheless, high titer sera from mice immunized with only the GP protein did not neutralize EBOV. This may relate to the quality of the protein antigens, and the absence of neutralizing antibodies in this group can most likely be attributed to the exposure of mainly non-neutralizing GP epitopes present on non-native GP. However, other studies have demonstrated the feasibility of using protein antigens to induce protective immunity in small animal models^48^, and our study was not designed to evaluate the merits of a homologous purified GP protein prime-boost vaccine approach. Importantly, one priming immunization with a DREP construct was sufficient to direct the protein boosted antibody responses to neutralizing epitopes. This highlights the importance of being able to assay anti-Ebolavirus antibody responses in a functional assay, and it might therefore be informative to complement neutralization assays with ADCC or CDC assays. In fact, it has been suggested that antibodies that are associated to ADCC or CDC activity are important for protection against Ebolavirus infection^33,34,48^. In our study, D-GP immunization promotes IgG2a over IgG1production, which should favor such cytotoxic activity. In general, DREP appears to favor IgG2a induction over IgG1. In this study, the IgG2a polarization was more prominent than previously reported from DREP constructs expressing HIV-1 gp140^32^. In contrast to this study, the DREP-gp140 was a relatively poor inducer of antibody responses, but elicited vigorous CD8 + T cell responses. Thus, it appears as both antigen and vector contribute to the Th1 profile elicited by DREP vaccines encoding viral glycoproteins.

In this study, we also report that a heterologous boost with an MVA-based viral vector expressing GP further increased the antibody titers and should correlate to increased potency of the vaccine regimen. This is consistent with findings of other investigators^49^. In particular, MVA provided a very strong boost to the ChAd3-EBOZ vaccine in a phase I clinical trial^50^. The MVA-GP generated for this study takes advantage of an optimized parental MVA with deletions of three immunomodulatory vaccinia genes (*C6L*, *K7R* and *A46R*), and thus differ from other MVA-based vaccines against Ebolavirus. Such deletions have previously been reported to improve the potency of the MVA-based vaccine candidates^51,52^. Thus, these optimized MVA-GP vaccine candidates could be used in further studies in NHP or human clinical trials together with DREP-GP for eliciting high, broad and protective Ebolavirus-specific immune responses.

Several investigators have reported that expression of GP and VP40 Ebolavirus proteins in eukaryotic cells generate filovirus-like particles, whereas expression of GP generates pleomorphic particles^29,53^. We hypothesized that our vaccines could differ in immunogenicity due to these different properties, and that the GP-VP40 might be more immunogenic because of its production of particles having a “natural” Ebolavirus like shape. However, despite the presence of VLP in cell supernatants, the production of filovirus-like structures by D-GP-VP40 did not improve immunogenicity. On the contrary, the GP-VP40 constructs were less immunogenic than constructs expressing GP alone. While this manuscript was in preparation, a paper was published using a similar DREP-based approach expressing the EBOV GP and VP40 but from separate constructs^54^. That study showed that inclusion of the DREP-VP40 in the vaccine cocktail did not improve antibody responses compared to responses generated by an EBOV GP only vaccine. The authors describe Filovirus-like particle production in cells co-transfected with both plasmids *in vitro*, but it remains unclear at what frequency co-transfection occurred *in vivo*. No analysis of virus neutralization was performed to detect possible qualitative differences. Our D-GP-VP40-Z constructs elicited antibody responses of equivalent endpoint titers as compared to those reported by Ren *et al*. However, immunization with DREP-GP alone elicited significantly higher responses in our study. This may suggest that co-expression of VP40 could have a suppressive effect on antibody responses to GP. The Marburg virus VP40 N-terminal domain, which shares substantial homology with Ebolavirus VP40, has been shown to suppress immune signaling in infected cells by antagonizing Jak and STAT tyrosine phosphorylation thus inhibiting type I and III IFN-induced gene expression^55,56^. Hypothetically, a similar role for Ebolavirus VP40 may have contributed to the decreased immune responses against GP in both studies.

In our studies we show that a bivalent vaccine can elicit Ebolavirus species-specific antibody responses. It will be interesting for future studies to include DREP encoding GP from additional Ebolavirus species and even other filoviruses such as Marburg virus. Moreover, if new Ebolavirus species emerge, a vaccine platform that can be rapidly adapted to emerging viruses is highly desirable.

The DREP platform evaluated in this study has the advantage that it is inexpensive, safe, and easy to produce. The platform also lends itself to rapid construction such that potential new variants of Ebolavirus in an emerging outbreak can be rapidly targeted^17^. Thus, for re-emerging pathogens like Ebolavirus, the DREP platform can be an efficient and cost-effective way to replace the traditional large-scale antigen production or technology platforms that require extended time for implementation.

## Methods

### DREP Ebolavirus vaccine candidates

DREP Ebolavirus vaccine constructs were made by cloning the GP or GP-2A-VP40 genes of EBOV Mayinga (AF086833.2) and SUDV Boniface (FJ968794.1) variants into the Semliki Forest Virus (SFV) DREP plasmid vector backbone^15,16,32^ (Fig. 1). GP and VP40 genes were codon optimized for human expression and synthesized (GeneArt) and GP was edited (adding an additional A residue at the natural RNA editing site) to render the full-length GP protein and to avoid the presence of the secreted GP protein. The GP-2A-VP40 fusion gene was separated by a Foot-and-Mouth Disease Virus (FMDV)-derived 2A-like sequence that promotes ribosomal skipping, which results in production of both GP and VP40 as individual proteins from the same open reading frame. Constructs were confirmed by sequencing and plasmid DNA of the different DREP Ebolavirus vaccine candidates was purified from bacterial cultures using the EndoFree Plasmid Maxi Kit (QIAGEN).

Recombinant GP-Sudan and GP-Zaire proteins (P-GP-S and P-GP-Z) were used for immunizations and as ELISA antigens (Icosagen). The GP Sudan and Zaire sequences were based on the GP consensus sequence of the respective viruses. P-GP-Z was based on isolates of the 2014 Ebola outbreak, and had 100% identity with many late isolates, e.g. KM034549.1. P-GP-S was based on all GP Sudan sequences in NCBI nucleotide and protein databases available in September 2014. The consensus was biased to later sequences and had >99% identity to 2012 outbreak isolates of SUDV, 94% to Boniface SUDV and >99% to AGL73425.1. The recombinant proteins were expressed in HEK-293 cells as GP preprotein with the transmembrane domain deleted resulting in secretion of soluble trimerized GP1-GP2 heterodimers that were purified using a His6 tag placed into C-terminus of GP2.

### Construction of recombinant MVA-GP vaccine candidates

MVA-GP vaccine candidates (MVA-GP Zaire and MVA-GP Sudan) were constructed by inserting the GP of EBOV Mayinga or SUDV Boniface variants into the TK locus of the MVA genome as previously described^52,57^. The MVA strain used as the parental vector for the generation of the recombinant MVA-GP vaccine candidates is an optimized MVA containing immunopotentiating deletions in the vaccinia immunomodulatory genes *C6L*, *K7R* and *A46R*^51,52^. GP expression is under the transcriptional control of the viral synthetic early/late promoter. MVA-GP Zaire and MVA-GP Sudan were grown in chicken embryo fibroblast (CEF) cells and purified through two 36% (wt/vol) sucrose cushions. The correct insertion of the GP genes was confirmed by PCR and sequencing and the GP expression was analyzed by western blotting.

### *In vitro* GP and VP40 expression analysis by western blot and electron microscopy

BHK-21 cells were transfected using Lipofectamine 3000 (Invitrogen) according to the manufacturer’s instructions. Cell lysates were harvested 24 h later using ice-cold lysis buffer (20 mM HEPES pH 7.4; 110 mM potassium acetate; 2 mM magnesium chloride; 0.1% Tween 20; 1.0% Triton X-100, 0.5% sodium deoxycholate, 0.5 M sodium chloride and Protease Inhibitors cocktail), and cell debris was removed by centrifugation at 4 °C for 10 min at 14,000 rpm. Samples were then diluted in 4x Laemmli loading buffer (Bio-Rad), and run on a 12.5% SDS-PAGE before proteins were transferred onto a nitrocellulose membrane. Expression was detected using mouse anti-GP SUDV and EBOV antibodies (IBT Bioservices), mouse anti-VP40 SUDV antibody (IBT Bioservices), rabbit anti-VP40 EBOV antibody (IBT Bioservices) and appropriate secondary horseradish peroxidase (HRP) conjugated anti-Ig antibodies (Sigma). Ebolavirus VLPs or recombinant Ebolavirus VP40 protein were used as positive controls, and non-transfected BHK-21 cells as negative control. For detection of VLP formation from the EBOV and SUDV GP and GP-2A-VP40 replicons, RNA was packaged into virus particles using the standard split helper system, as previously described^58^. These particles were used to efficiently deliver replicon RNA into BHK-21 cells. Cell culture supernatants were collected and VLPs were purified from supernatants, concentrated by ultracentrifugation, and frozen until EM analysis. A drop of the VLP suspension was incubated on a Formvar/Carbon-coated copper grid (Axlab) for at least 1 minute. The sample was then stained with 2% phosphotungstic acid (VWR Prolabo), pH 6. The sample was examined with a transmission electron microscope (Tecnai TM G2 Spirit bioTWIN) at a magnification ranging from 30,000 to 96,000x.

### Mice and Immunizations

Female 7–12 weeks old BALB/c mice were purchased from Charles River. Animals were kept at the department of Microbiology, Tumor and Cell Biology (MTC) in accordance with the recommendations of the Swedish Board of Agriculture. The protocol was approved by the local ethics committee, Stockholms norra djurförsöksetiska nämnd, permit number N82/14.

Prior to immunization all mice were anesthetized by isoflurane inhalation. DREP vaccines were administered by intradermal (i.d.) needle injection followed by electroporation (EP), as has been described previously^16^. A total volume of 2 × 20 µl was injected i.d. containing 5 µg DREP DNA with a 30-gauge insulin syringe (BD Micro-Fine). Then, a needle array electrode with two parallel rows of four 2 mm pins was placed at the injection spot, and 2 pulses of 1.125 V/cm for 50 µs followed by 8 pulses of 275 V/cm for 10 ms were applied.

The recombinant GP SUDV and EBOV proteins (5 µg of each) were formulated in the adjuvant Monophosphoryl Lipid A (MPLA, Polymun) at a 2:1 protein to adjuvant mass ratio 30 min before immunzation and administered intramuscularly (i.m.) 50 μl in each hind leg *gastrocnemius* muscle.

10^7^ plaque forming units (pfu) of MVA-GP Zaire or MVA-GP Sudan were delivered intraperitoneally (i.p.) diluted in 100 μl of PBS.

### ELISA

Sera were collected from blood samples after centrifugation at 8500 × g for 8 min at room temperature. ELISA plates (Immunosorp, Nunc) were coated with 2 µg/ml (50 µl/well) of SUDV or EBOV GP proteins (Icosagen) diluted in PBS, and incubated at 4 °C overnight. Plates were washed three times with PBS-Tween (0.05%) and then blocked with 1% BSA in PBS (100 µl/well) for 2 h at 4 °C. Sera were diluted in PBS-Tween (0.05%) at a 1:100 starting dilution and then serially diluted in six three-fold dilution steps until a 1:72900 dilution (50 µl/well). Plates were then incubated at 4 °C overnight, and washed three times with PBS-Tween before a HRP-conjugated anti mouse-IgG (Southern Biotech) was added at a 1:5000 dilution in PBS-Tween (50 µl/well). After 1.5 h incubation at room temperature, plates were washed five times with PBS-Tween, before addition of 50 µl of o-phenylenediamine dihydrochloride substrate (Sigma fast, Sigma-Aldrich). After 15 min at room temperature, the reaction was stopped with 25 µl 1 M HCl and the optical density (OD) was read at 490 nm using an ELISA reader. For calculation of endpoints titers, a cutoff value based on the OD values from the naïve controls was determined (average from naïve control sera + 2 standard deviations). Titers were determined by interpolating the point where sigmoid curve reaches the cutoff value using the GraphPad Prism 7 software.

### Neutralization assay

VeroE6 cells were grown in Eagles Modified Essential medium (EMEM) with 5% fetal calf serum, 2 mM L-glutamine, 0.3% NaHCO_3_ and 0.5% PEST. Sera were incubated at 56 °C for 60 min to inactivate the complement system, and diluted in medium in a 2-fold dilution series starting at a 1:10 dilution. Each serum dilution was mixed with 50 pfu of EBOV (p3d9 141030 ML/CA virus) and incubated for 1 h before distribution to the plates in duplicates. As positive control, triplicates wells were infected with 100 pfu, 50 pfu, 25 pfu and 12.5 pfu. A virus-free negative control was also included. Cells were analyzed with a microscope for cytopathic effect (CPE) every three days until all cells in the virus control showed a clear CPE. Neutralization titers were calculated as the highest serum dilution with a full neutralizing capacity (absence of CPE) in both duplicates.

### Isolation of splenocytes

Fresh mouse spleens were mashed though 70-µm cell strainers. Cells were then washed in complete RPMI medium [RPMI 1640 supplemented with 5% fetal bovine serum (FBS), 2 mM L-glutamine, 100 U/ml penicillin and 100 µg/ml streptomycin] (Thermo Fisher Scientific) and collected by centrifugation (350 × g at room temperature). The cell pellet was resuspended in 1 ml of red blood cell lysis buffer (Sigma-Aldrich) for 2 min. Lysis was stopped by addition of complete RPMI, and the cells were centrifuged as above before they were resuspended in complete RPMI medium. Cells were quantified with the Countness II™ automated cell counter (Thermo Fisher Scientific).

### IFN-γ ELISpot assay

Multiscreen-IP plates (Millipore) were activated, coated and blocked according to the kit manufacturer’s instructions (Mabtech). 2 × 10^5^ freshly isolated splenocytes were added per well in triplicates with either 2 µg/ml the LYDRLASTV peptide present in both SUDV and EBOV GP^38^, or the RPHTPQFLF peptide derived from SUDV GP^38^, medium alone or Concanavalin A (Sigma-Aldrich). After 20 ± 2 h of incubation at 37 °C with 5% CO_2_, plates were developed as recommended by the manufacturer (Mabtech). Plates were analyzed using the Immunospot analyzer and software (Immunospot).

### Statistical analyses

GraphPad Prism 7 software (GraphPad Software Inc.) was used for statistical analyses. A P-value < 0.05 was considered significant (*P < 0.05; **P < 0.01; ***P < 0.001; ****P < 0.001).

## Electronic supplementary material

Supplementary Figure 1

## Data Availability

All data generated or analyzed during this study are included in this article.
