# Supplementary material for: DNA-launched RNA replicon vaccines induce potent anti-Ebolavirus immune responses that can be further improved by a recombinant MVA boost
Source: Sci Rep. 2018 Aug 20;8:12459. doi: 10.1038/s41598-018-31003-6 (PMC6102224; doi:10.1038/s41598-018-31003-6)
Supplement: Supplementary file 1 — Supplementary Figure 1 [file 41598_2018_31003_MOESM1_ESM.pdf]

DNA-launched RNA replicon vaccines induce potent anti-Ebolavirus immune responses that can be further improved by a recombinant MVA boost

Running title: Replicon vaccines induce anti-Ebolavirus immune responses

Pontus Öhlund[1]#, Juan García-Arriaza[2], Eva Zusinaite[3], Inga Szurgot[1], Andres Männik[4], Annette Kraus[5], Mart Ustav[4], Andres Merits[3], Mariano Esteban[2], Peter Liljeström[1], and Karl Ljungberg[1*]

1. Department of Microbiology, Tumor and Cell Biology, Karolinska Institutet, Stockholm, Sweden.
2. Department of Molecular and Cellular Biology, Centro Nacional de Biotecnología, Consejo Superior de Investigaciones Científicas (CNB-CSIC), Madrid, Spain.
3. Institute of Technology, University of Tartu, Tartu, Estonia.
4. Icosagen Cell Factory OÜ, Ülenurme vald, Tartumaa, Estonia.
5. Department of Microbiology, Public Health Agency of Sweden, Solna, Sweden.
#. Present address: Department of Biomedical Science and Veterinary Public Health; Virology Unit, Swedish University of Agricultural Sciences, Uppsala, Sweden

*. Address correspondence to Karl Ljungberg, Karl.Ljungberg@ki.se

P.Ö., J.G.A. and E.Z. contributed equally to this work.

Supplementary figure 1.

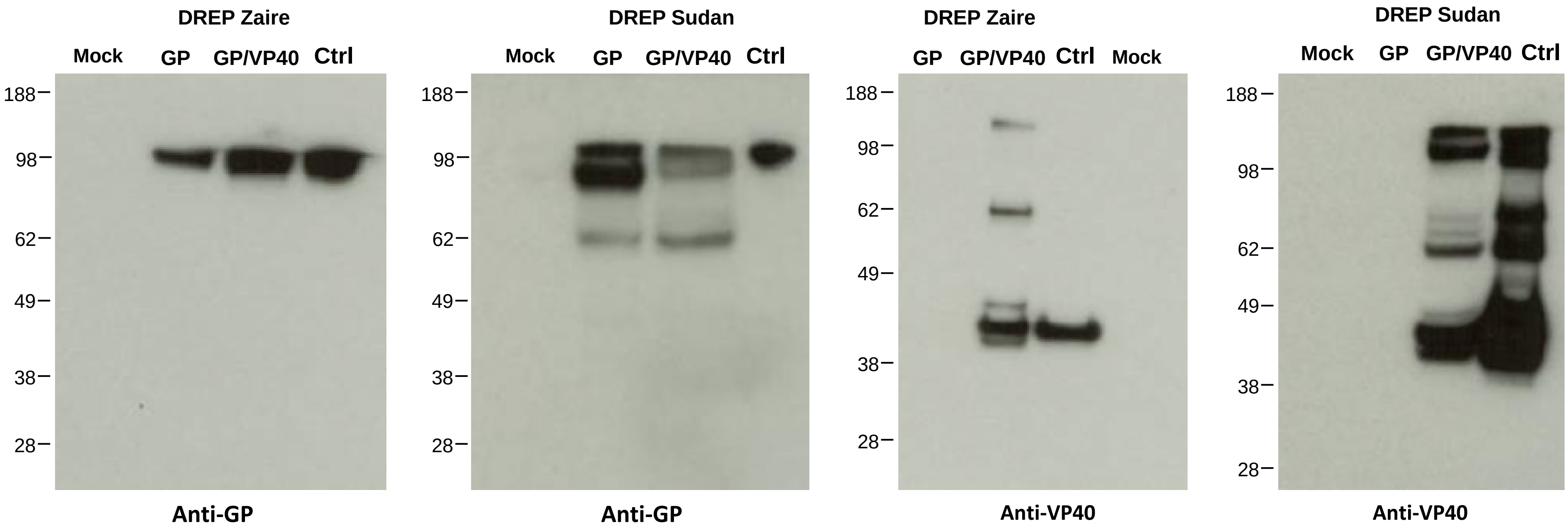

Supplementary figure 1. Analysis of Ebolavirus GP and VP40 expression. Full-length (uncropped) blots presentation. BHK-21 cells were transfected using Lipofectamine 3000 (Invitrogen) according to the manufacturer's instructions. Cell lysates were harvested 24h later using ice-cold lysis buffer (20 mM HEPES pH 7.4; 110 mM potassium acetate; 2 mM magnesium chloride; 0.1% Tween 20; 1.0% Triton X-100, 0.5% sodium deoxycholate, 0.5 M sodium chloride and Protease Inhibitors cocktail), and cell debris was removed by centrifugation at 4°C for 10 min at 14,000 rpm. Samples were then diluted in 4x Laemmli loading buffer (Bio-Rad), and run on a 12.5% SDS-PAGE before proteins were transferred onto a nitrocellulose membrane. Expression was detected using mouse anti-GP SUDV and EBOV antibodies (IBT Bioservices), mouse anti-VP40 SUDV antibody (IBT Bioservices), rabbit anti-VP40 EBOV antibody (IBT Bioservices) and appropriate secondary horseradish peroxidase (HRP) conjugated anti-Ig antibodies (Sigma). For the detection of HRP, Pierce ECL Western Blotting Substrate was used. Blots were then exposed to X-ray film and developed using AGFA Currix 60 processor after 30s exposure. Ebolavirus VLPs or recombinant Ebolavirus VP40 protein were used as positive controls, and non-transfected BHK-21 cells as negative control. No image processing (such as changing brightness and contrast) has been applied.
